# Digital breast tomosynthesis (DBT): recommendations from the Italian College of Breast Radiologists (ICBR) by the Italian Society of Medical Radiology (SIRM) and the Italian Group for Mammography Screening (GISMa)

**DOI:** 10.1007/s11547-017-0769-z

**Published:** 2017-05-25

**Authors:** Daniela Bernardi, Paolo Belli, Eva Benelli, Beniamino Brancato, Lauro Bucchi, Massimo Calabrese, Luca A. Carbonaro, Francesca Caumo, Beatrice Cavallo-Marincola, Paola Clauser, Chiara Fedato, Alfonso Frigerio, Vania Galli, Livia Giordano, Paolo Giorgi Rossi, Paola Golinelli, Doralba Morrone, Giovanna Mariscotti, Laura Martincich, Stefania Montemezzi, Carlo Naldoni, Adriana Paduos, Pietro Panizza, Federica Pediconi, Fiammetta Querci, Antonio Rizzo, Gianni Saguatti, Alberto Tagliafico, Rubina M. Trimboli, Marco Zappa, Chiara Zuiani, Francesco Sardanelli

**Affiliations:** 1 U.O. Senologia Clinica e Screening Mammografico, Dipartimento di Radiologia, APSS, Centro per i Servizi Sanitari, Pal. C, viale Verona, 38123 Trento, Italy; 2 Dipartimento di Scienze Radiologiche, Università Cattolica del Sacro Cuore, Largo Agostino Gemelli 8, 00168 Rome, Italy; 3 Zadig Scientific Communication Agency, Via Arezzo 21, 00161 Rome, Italy; 4 Struttura Complessa di Senologia Clinica, Istituto per lo Studio e la Prevenzione Oncologica (ISPO), Via Cosimo il Vecchio 2, 50139 Florence, Italy; 5 Romagna Cancer Registry, Romagna Cancer Institute (IRST) IRCCS, Via Piero Maroncelli 40, Meldola, 47014 Forlì, Italy; 6 UOC Senologia Diagnostica, IRCCS AOU San Martino-IST, Largo Rosanna Benzi 10, 16132 Genoa, Italy; 7 Unit of Radiology, IRCCS Policlinico San Donato, Via Morandi 30, San Donato Milanese, 20097 Milan, Italy; 8 UOSD Breast Unit ULSS 20, Piazza Lambranzi 1, 37142 Verona, Italy; 9 Dipartimento di Scienze Radiologiche, Oncologiche ed Anatomo-patologiche, Policlinico Umberto I, Sapienza Università di Roma, Viale Regina Elena 324, 00161 Rome, Italy; 10 Division of Molecular and Gender Imaging, Department of Biomedical Imaging and Image-guided Therapy, Medical University of Vienna/General Hospital Vienna, Waehringer Guertel 18-20, 1090 Vienna, Austria; 11 Regional Screening Coordinating Centre, Veneto Region, Venice, Italy; 12 Regional Reference Centre for Breast Cancer Screening, Turin, Italy; 13 Mammography Screening Centre, Local Health Authority, Modena, Italy; 14 Epidemiology Unit, Centre for Cancer Prevention, Turin, Italy; 15 Interinstitutional Epidemiology Unit, AUSL Reggio Emilia, and Arcispedale S. Maria Nuova, Reggio Emilia, Italy; 16 Medical Physics Service, Local Health Authority, Modena, Italy; 17 Radiologia 1U, Dipartimento di Diagnostica per Immagini, Università di Torino, A. O. U. Città della Salute e della Scienza di Torino, Via Genova 3, 10126 Turin, Italy; 18 U.O. Radiodiagnostica, Candiolo Cancer Institute, FPO, IRCCS, Strada Provinciale 142, km 3.95, Candiolo, 10060 Turin, Italy; 19 DAI Patologia e Diagnostica, Azienda Ospedaliera Universitaria Integrata, Piazzale A. Stefani 1, 37126 Verona, Italy; 20 Department of Health, Emilia-Romagna Region, Bologna, Italy; 21 U.O. Radiologia Senologica, IRCCS Ospedale San Raffaele, Via Olgettina 60, 20132 Milan, Italy; 22 Department of Prevention, Screening Centre, Local Health Authority, Sassari, Italy; 23 Pathology Department, Local Health Authority, Asolo, Treviso, Italy; 24 Senology Unit, Local Health Authority, Bologna, Italy; 25 Department of Experimental Medicine, DIMES, Institute of Anatomy, University of Genova, Via de Toni 14, 16132 Genoa, Italy; 26 Department of Biomedical Science for Health, Università degli Studi di Milano, Via Mangiagalli 31, 20133 Milan, Italy; 27 UOC Epidemiologia Clinica, Istituto per lo Studio e la Prevenzione Oncologica (ISPO), Florence, Italy; 28 Institute of Radiology, University of Udine, Piazzale S. M. della Misericordia 15, 33100 Udine, Italy; 29 Department of Biomedical Sciences for Health, Università degli Studi di Milano, Via Morandi 30, San Donato Milanese, 20097 Milan, Italy

**Keywords:** Breast cancer, Digital breast tomosynthesis, Mammography, Screening

## Abstract

This position paper, issued by ICBR/SIRM and GISMa, summarizes the evidence on DBT and provides recommendations for its use. In the screening setting, DBT in adjunct to digital mammography (DM) increased detection rate by 0.5–2.7‰ and decreased false positives by 0.8–3.6% compared to DM alone in observational and double-testing experimental studies. The reduction in recall rate could be less prominent in those screening programs which already have low recall rates with DM. The increase in radiation exposure associated with DM/DBT protocols has been solved by the introduction of synthetic mammograms (sDM) reconstructed from DBT datasets. Thus, whenever possible, sDM/DBT should be preferred to DM/DBT. However, before introducing DBT as a routine screening tool for average-risk women, we should wait for the results of randomized controlled trials and for a statistically significant and clinically relevant reduction in the interval cancer rate, hopefully associated with a reduction in the advanced cancer rate. Otherwise, a potential for overdiagnosis and overtreatment cannot be excluded. Studies exploring this issue are ongoing. Screening of women at intermediate risk should follow the same recommendations, with particular protocols for women with previous BC history. In high-risk women, if mammography is performed as an adjunct to MRI or in the case of MRI contraindications, sDM/DBT protocols are suggested. Evidence exists in favor of DBT usage in women with clinical symptoms/signs and asymptomatic women with screen-detected findings recalled for work-up. The possibility to perform needle biopsy or localization under DBT guidance should be offered when DBT-only findings need characterization or surgery.

## Introduction

In the last years, many reports have appeared about digital breast tomosynthesis (DBT) in both the diagnostic and screening setting. In fact, thanks to a pseudo-three-dimensional reconstruction, DBT allows for overcoming some limitations of standard two-dimensional (2D) digital mammography (DM) caused by structural overlapping and resulting into false-negative and false-positive findings [1]. The X-ray tube moves along an angular direction and generates multiple low-dose variably angled projections of the compressed breast. The reconstruction of multiple images of thin slices of the compressed breast is obtained by specialized software [2, 3]. The technical solutions proposed by vendors differ for tube movement (continuous/discontinuous), width of oscillation angle, number of projections, type of detector, and other characteristics, although no substantial effects on diagnostic performance related to these differences have been reported so far.

This position paper, issued by the Italian College of Breast Radiologists by the Italian Society of Medical Radiology (SIRM) and the Italian Group for Mammography Screening (GISMa), summarizes the available evidence on DBT and provides practical recommendations for its use. For the levels of evidence (LoE) reported here, we refer to the definitions given by the Centre for Evidence-Based Medicine, Oxford, United Kingdom [4].

## DBT for first-level screening

Several studies have evaluated the potential of DBT in first-level screening [5–14]. In particular, three European prospective trials conducted in the context of organized population-based screening programs [6–9] and five retrospective studies from spontaneous screening settings [10–14] have shown that DBT combined with DM (or as a stand-alone approach [9]) allows for a better diagnostic performance than DM alone.

Results of DBT differ according to the study design, the screening interval, and the type of screening setting (organized versus spontaneous). A review [15] reported that DBT provides an increase in cancer detection rate from 0.5 to 2.7 per thousand screened women and a reduction in false-positive recall rate from 0.8 to 3.6 per 100 screened women. The possibility of using only the mediolateral oblique DBT projection, supported by one study [9], has the rationale of dose reduction. However, although many experiences have shown the superior diagnostic performance of two-view DBT compared with one-view DBT [16–18], the availability of synthetic 2D mammograms generated from the DBT dataset (see below) has substantially overcome the problem of dose. A reduction in recall rate of greatly varying magnitude (from 6 to 82%, median 31%) has been found [19]. Notably, this variability depends on the baseline recall rate with standard DM because the higher the baseline DM recall rate, the higher the absolute and relative reduction obtained with DBT. Thus, the advantage of reducing the recall rate could be less prominent in those screening programs which already have low recall rates with standard DM.

If both DM and DBT are acquired (separately or with the so-called “combo” modality), the average glandular dose is approximately doubled. In fact, the DBT dose is similar to that of DM [20–22]. However, considering the dose reduction obtained through the introduction of DM as an alternative to film-screen mammography, the dose of these DM/DBT protocols too remains below the upper limit defined by the “European guidelines for quality assurance in breast cancer screening and diagnosis” published in 2006 [23].

Of note, radiation exposure would be an issue to take into consideration for a generalized adoption of DM/DBT protocols for population-based mass screening. The solution has come from the synthetic digital mammography (sDM) obtained through specialized algorithms summing and filtering the DBT datasets. Principles and methods have similarities with those generating maximum intensity projections in computed tomography and magnetic resonance imaging (MRI). These sDMs are virtual 2D mammograms obtained from DBT, paying no tradeoff in terms of radiation exposure [24–26].

Several authors have compared sDM/DBT protocols with DM/DBT protocols demonstrating a similar diagnostic performance [25–28]. Therefore, unless additional radiation exposure is specifically justified (as in the work-up of suspicious findings detected at DM), a diagnostic mammography examination can be performed using the sDM/DBT approach.

Notably, two studies [29, 30] have reported that 54–57% of additional cancers detected by additional ultrasonography screening after negative DM were detected by DBT. This is a relevant argument in favor of DBT, considering the practical hurdles for a generalized mass screening with ultrasonography in adjunct to DM.

However, in the context of organized population-based screening programs, a simple increase in sensitivity and overall diagnostic performance of a new tool, even if statistically significant and clinically relevant, is not enough, per se, for its generalized adoption. According to the European Council Recommendation on cancer screening [31], evidence from randomized controlled trials is needed before introducing new screening tools. In particular, considering the pre-existing evidence in favor of screening mammography (recently confirmed by the International Agency for Research on Cancer [32]), caution is urged due to the possibility that a substantial part of the additional cancers detected by DBT could be overdiagnosed lesions, i.e. indolent malignant lesions that would never surface clinically during the woman’s life [33]. Importantly, the consequence would be an increase in overtreatment, i.e. unnecessary surgery, radiation, and/or medical therapy.

It is worth to note that most of the additional cancers detected with DBT have been reported to be invasive [6–14]. A large study from the United States [10] has compared DM/DBT in approximately 174,000 women with DM alone in approximately 281,000 women. The DM/DBT approach was associated with a 29% increase in detection rate and a 15% reduction in recall rate. All the increase in cancer detection was due to invasive cancers (+41%). No increase in the detection of ductal carcinoma in situ, deemed more probably overdiagnosed, was observed. However, we should consider that also invasive cancers can be indolent and overdiagnosed. Thus, no definitive conclusions can be drawn.

Several other issues cannot be ignored when discussing the potential introduction of DBT for screening. They include at least sufficient availability of DBT equipments, management of suspicious findings at DBT alone, and increased reading time [34–36], which implies the need for more breast radiologists involved as screening readers.

Thus, before introducing DBT as a first-level screening tool, we should wait for the results of randomized controlled trials (some of which are already ongoing in Italy) comparing a population sample having DM/DBT or sDM/DBT at the first round and DM alone at the subsequent round with a population sample having DM alone at both rounds [37]. This will enable us to determine:

1. whether the expected increase in cancer detection at first round with DBT is associated or not associated with a reduction in interval cancer incidence;
2. whether the total incidence of advanced cancers (stage ≥T2) in the whole study period (i.e. considering all cancers detected in the first or second round as well as interval cancers) is lower in the DBT group compared with the control group; and
3. whether the total incidence of breast cancer in the DBT group exceeds that of the control group.

The attention paid to interval cancer rate is explained by the possibility to use this measure as a proxy of effectiveness in mortality reduction [15]. To our knowledge, only two articles [38, 39] have reported results concerning the interval cancer rate of DBT screening. The study design was a single-center retrospective analysis [38] and a multicenter [39] prospective cohort study. Comparing DM alone with DM/DBT screening, a reduction in interval cancer rate was observed in both studies, from 0.7 to 0.5 per thousand screened women [38] and from 0.6 to 0.46 per thousand screened women [39], respectively. These variations were not statistically significant. However, it should be noted that neither study was designed and powered for interval cancer analysis, and that both were conducted in a spontaneous screening setting. These heavy limitations prevent us to draw any conclusion to be extrapolated to European organized biennial screening programs.

## DBT for work-up of screen-detected suspicious findings, as a first-line diagnostic examination in symptomatic women, for preoperative staging, and targeted evaluation after MRI

Several studies [40–48] have shown that DBT is at least equivalent to additional DM views (magnification, spot compression, 90° views, etc.), also reducing radiation exposure in both the diagnostic and screening setting. In symptomatic women, diagnostic accuracy is improved by DBT, reducing the number of suspicious findings and of unnecessary biopsies [43, 45, 46]. In the presence of palpable lesions, DBT has been reported to be never worse, and often better, than DM for estimating tumor size [49]. The better diagnostic performance of DBT is confirmed also in the case of invasive lobular cancers, as shown by a recent study [50]. In the preoperative setting, the combination of DM, DBT, and ultrasonography could provide the same information as MRI [51]. Finally, DBT allows for identifying some findings additionally found on preoperative MRI also when they are not visible at targeted ultrasonography, permitting a reduction in the number of MR-guided biopsies [52].

Studies evaluating inter-reader variability for DM/DBT versus DM alone have reported for each reader an increase in the detection rate and a reduction in the recall rate with an overall increase in the diagnostic performance [53–56]. In the particular context of spontaneous screening, DBT has also been found to reduce the number of short-time repeat examinations [55].

All these results allow for recommending DBT for symptomatic women and for work-up of screen-detected suspicious findings.

## Recommendations

Recommendations are presented here for five categories of women:

1. asymptomatic women at average risk (first level of organized or spontaneous screening);
2. asymptomatic women at intermediate risk, including women with a previous breast cancer;
3. symptomatic women and women needing work-up of screen-detected suspicious findings;
4. asymptomatic women at hereditary/familial high risk;
5. asymptomatic women at high risk due to previous chest radiotherapy.

A sixth, final recommendation concerns needle biopsy under DBT guidance.

1. Asymptomatic women at average risk (first level of organized or spontaneous screening)

These women should undergo DM starting not before the age of 40, with a screening interval that may vary according to local health authority’s decision. As recently stated by the European Society of Breast Imaging [57, 58], direct DM should be preferred to film-screen mammography due to a reduced radiation exposure [59] and an at least equivalent diagnostic performance [60] (LoE A). The preference is also in favor of DM when compared with indirect digital phosphor storage plate (so-called computer radiography) [61].

DBT can be used as a first-level screening tool in women at average risk:

- in the context of studies approved by an Ethical Committee, with enrollment after informed consent signature by the woman (for randomized controlled trials, refer to the scheme proposed by the Osservatorio Nazionale Screening [37]);
- in the well-defined context of centers being part of public population-based screening programs, with previous experience with ethically approved studies concerning at least feasibility of screening DBT, demonstrated through articles published in peer-reviewed journals.

In both cases, the sDM/DBT approach should be preferred (Loe A). In both cases, centers are requested to provide data regarding the screening process, service, and impact, including the women’s compliance. Considering the data management needs, the necessary interactions with information technology services, and the impact of DBT on the whole multidisciplinary team, studies should be performed under umbrella of breast units. Moreover, usual monitoring data should be collected, in particular absolute and/or proportional incidence of interval cancer and of screen-detected cancers of stage T2 or higher [37, 62, 63]. Attention should be paid to avoid that the implementation of DBT studies causes a reduction in screening coverage, compliance, or quality indicators.

2. Asymptomatic women at intermediate risk, including those with a previous breast cancer

Up to now, no substantial evidence has been produced in favor or against the use of DBT for screening women at intermediate risk of breast cancer, i.e. those with a lifetime risk from 15 to 19%, estimated using multifactorial models. All these women should undergo screening DM with the same protocols recommended for asymptomatic women at average risk.

For asymptomatic women with a previous history of breast cancer (included in this general category of women at intermediate risk), we refer to the recommendations provided by the GISMa and the Italian College of Breast Radiologists by SIRM [64]. The recent observation of a reduction in indeterminate findings in surveillance after breast cancer treatment [65] plays in favor of DBT usage (LoE D) in this setting, with a preference for sDM/DBT protocols.

DBT can be used as a screening tool in women at intermediate risk:

- in the context of studies approved by an Ethical Committee, with enrollment after informed consent signature by the woman;
- in the well-defined context of centers having previous experience with ethically approved studies concerning DBT, demonstrated through articles published in peer-reviewed journals.

Usual performance indicators should be collected, in particular absolute and/or proportional incidence of interval cancer and of screen-detected cancers of stage T2 or higher [36, 61, 62].

3. Symptomatic women and women needing work-up of screen-detected suspicious findings

We include in this category both women with suspicious clinical symptoms or signs (asking for a mammogram usually ordered by a general practitioner or a specialist) and asymptomatic women with screen-detected suspicious findings recalled for work-up in the context of spontaneous or organized screening. Women with previous history of breast cancer, if having suspicious clinical symptoms/signs or after a suspicious finding on an imaging study, are also included in this category.

In these women, if an indication to mammography exists and DBT is available, DBT should be performed (LoE A), preferably with a sDM/DBT protocol (LoE B). If sDM mammograms are not available, DBT can be performed after DM (LoE B).

4. Asymptomatic women at hereditary/familial high risk

Women at hereditary/familial high risk should be screened in the context of dedicated pathways [66–71]. Considering the higher radiosensitivity of their breast tissue and the high sensitivity of MRI for breast cancer, mammography can be avoided at least up to 35 years of age, in particular in BRCA1 mutation carriers. If mammography is performed as an adjunct to MRI or in the case of MRI contraindications, a sDM/DBT protocol is suggested (LoE D). In all cases of mammographic work-up, DBT should be preferred to additional DM projections (LoE D).

5. Asymptomatic women at high risk due to previous chest radiotherapy

Recommendations regarding breast cancer screening for these women (mainly lymphoma survivors) were recently provided by the Italian College of Breast Radiologists by SIRM [72]. Both DM and contrast-enhanced MRI should be performed annually due to the suboptimal sensitivity of each of the two techniques [73–76]. A sDM/DBT protocol could be preferred to DM alone (LoE D).

6. Needle biopsy under DBT guidance

DBT can show doubtful/suspicious findings without any clinical correlate and even undetectable on DM, sDM, or ultrasonography [5–8]. This may occur in both the screening and diagnostic settings. In those cases, when the DBT finding is neither detectable at targeted ultrasonography nor at DM review, a needle biopsy (and, when necessary, also presurgical localization) should be performed under DBT guidance. Importantly, DBT guidance offers important advantages in terms of shorter procedure duration and reduced radiation exposure [77, 78] (LoE B).

As a consequence, centers offering DBT should also offer DBT-guided interventions for DBT findings not otherwise identifiable. These interventions can be performed at the same center where DBT was done or at another center functionally connected with the first one. At present, in fact, few centers are equipped with the device for DBT-guided interventions.

## Conclusions

Evidence available for DBT allows to recommend its usage for all cases of symptomatic women and women needing work-up of screen-detected suspicious findings (considering both spontaneous and organized screening). In these settings, when available, sDM/DBT protocols should be preferred for symptomatic women.

Breast cancer screening using the current mammography technology has been demonstrated to be effective in reducing mortality from the disease. From this perspective, completing the shift from analog film-screen mammography and computed radiography systems, that are still in use, to direct digital mammography systems ranks first in the order of priorities.

A generalized adoption of DBT as a first-level screening tool should wait for a specific evidence, in particular for a statistically significant and clinically relevant reduction in interval cancer rate (hopefully associated with a reduction in advanced cancer rates). When this evidence will be available, both asymptomatic women at average and intermediate risk (including those with a previous breast cancer history) will be allowed to be screened with DBT on a routine basis. Accurate data collection will be necessary for many years in order to assess the overall impact of DBT technology in terms of mortality reduction and other efficacy/effectiveness indicators. For high-risk women, when a mammogram is indicated, a sDM/DBT protocol should be preferred.

The already existing evidence, which has been built with a non-negligible contribution of Italian breast radiologists, plays in favor of DBT. A trend for making DBT the mammography of the next future can be outlined. However, even in the United States, where FDA approved the use of some DBT devices in some cases for screening, the use of DBT is still quite limited. A recent survey [79] among the members of the Society of Breast Imaging reported that of 670 responders, only 200 (30%) use DBT although 62% of non-users have planned to equip themselves with DBT.

Studies and researches are still needed for a deeper evaluation of DBT, a relevant innovation in breast imaging. In particular, the risk of an increased overdiagnosis and also several organizational issues (including increased reading time) suggest to wait for a more conclusive evidence before adopting DBT in population-based screening, a position shared with other experts and medical or health authority bodies worldwide [15, 57, 58, 80, 81].
